# The importance of the timing of microbial signals for perinatal immune system development

**DOI:** 10.20517/mrr.2023.03

**Published:** 2023-04-15

**Authors:** Dale Archer, Maria Elisa Perez-Muñoz, Stephanie Tollenaar, Simona Veniamin, Christopher C. Cheng, Caroline Richard, Daniel R. Barreda, Catherine J. Field, Jens Walter

**Affiliations:** ^1^Department of Biological Sciences, University of Alberta, Edmonton, Alberta T6G 2R3, Canada.; ^2^Department of Agricultural, Food, and Nutritional Science, University of Alberta, Edmonton, Alberta T6G 2R3, Canada.; ^3^Department of Medicine, University of Alberta, Edmonton, Alberta T6G 2R3, Canada.; ^4^Center of Excellence for Gastrointestinal Inflammation and Immunity Research, University of Alberta, Edmonton, Alberta T6G 2R3, Canada.; ^5^APC Microbiome Ireland, School of Microbiology and Department of Medicine, University College Cork, Cork T12 YN60, Ireland.

**Keywords:** Gut microbiome, *Limosilactobacillus reuteri*, a window of opportunity, prenatal, postnatal, immune system, germ-free, probiotics

## Abstract

**Background:** Development and maturation of the immune system begin *in utero* and continue throughout the neonatal period. Both the maternal and neonatal gut microbiome influence immune development, but the relative importance of the prenatal and postnatal periods is unclear.

**Methods:** In the present study, we characterized immune cell populations in mice in which the timing of microbiome colonization was strictly controlled using gnotobiotic methodology.

**Results:** Compared to conventional (CONV) mice, germ-free (GF) mice conventionalized at birth (EC mice) showed few differences in immune cell populations in adulthood, explaining only 2.36% of the variation in immune phenotypes. In contrast, delaying conventionalization to the fourth week of life (DC mice) affected seven splenic immune cell populations in adulthood, including dendritic cells and regulatory T cells (Tregs), explaining 29.01% of the variation in immune phenotypes. Early life treatment of DC mice with *Limosilactobacillus reuteri* restored splenic dendritic cells and Tregs to levels observed in EC mice, and there were strain-specific effects on splenic CD4+ T cells, CD8+ T cells, and CD11c+ F4/80+ mononuclear phagocytes.

**Conclusion:** This work demonstrates that the early postnatal period, compared to the prenatal period, is relatively more important for microbial signals to influence immune development in mice. Our findings further show that targeted microbial treatments in early life can redress adverse effects on immune development caused by the delayed acquisition of the neonatal gut microbiome.

## INTRODUCTION

Development and maturation of the immune system begins *in utero* and continues throughout the neonatal period as the immune system encounters novel foreign antigens^[1-3]^. The immune system of neonates is typically characterized by a bias toward anti-inflammatory responses, tolerogenic responses, and suppressed activation of intestinal lymphoid cells^[2,3]^. This allows for the maturing immune system to develop tolerance to the abundance of novel foreign antigens from the environment, diet, and gut microbiome introduced in the neonatal period. Despite the established role of microbial signals for immune system development^[4-10]^, we are only beginning to understand the importance of the timing of microbial signals for immune development and the consequences of aberrations to this process in the perinatal period.

There is growing evidence of a critical window of opportunity in early life wherein the gut microbiome provides essential signals for the development of the host’s immune system^[11]^. For instance, studies using germ-free (GF) mice have demonstrated that certain immune cell populations and functions require microbial signals in the preweaning period for their development^[12-15]^. Moreover, recent studies have begun to elucidate the timing of this critical period for immune development and the underlying mechanisms. Knoop *et al.* demonstrated that the preweaning period is particularly important for interactions between the gut microbiome and the immune system, which are mediated by goblet cell-associated antigen passages in the intestinal epithelial layer^[16]^. These interactions were especially important for developing tolerance toward resident gut microbes^[16]^. Similarly, Al Nabhani *et al.* found that the gut microbiome in the preweaning period was important for inducing an immune reaction at weaning, termed the “weaning reaction,” which was essential for the maturation of the immune system and the development of tolerogenic responses^[17]^. Collectively, these studies provide evidence of an important period early in life for the induction of immune development by gut microbes that cannot be corrected later in life.

While the importance of the gut microbiome in early postnatal immune development is increasingly well understood and generally well accepted, less is known about the role of microbial signals in prenatal immune development. Recent studies have described viable microbial populations in human fetal tissues, including the intestines, and these microbes were associated with fetal immune development^[18,19]^. While there is much debate around the validity of these findings^[20-22]^ and the relevance of direct exposure of the fetus to microbes for prenatal immune development^[23]^, microbial metabolites from the maternal gut microbiome have been shown to modulate fetal development^[24-27]^. For instance, de Agüero *et al.* demonstrated that pups born to GF dams that were transiently colonized with *E. coli* HA107 during gestation (such that the dams were GF when the pups were born) had a greater abundance of intestinal NKp46+ RORγt+ group 3 innate lymphoid cells (ILCs) and CD11c+ F4/80+ mononuclear cell populations than pups born to untreated dams^[26]^. Furthermore, in humans, Li *et al.* identified a diverse set of microbially-derived metabolites in the intestines of second-trimester terminated fetuses, some of which were hypothesized to have a role in fetal immune system development^[28]^. Thus, it is increasingly recognized that microbial signals have an important role in immune development both prenatally and postnatally. However, the relative importance of the prenatal and early postnatal periods for microbial signals to modulate immune development has not been established.

Microbial-based strategies, such as probiotics, are increasingly being used to redress disruptions to immune development resulting from perturbations to neonatal gut microbiome assembly^[29-31]^. A promising candidate for this is *Limosilactobacillus reuteri*, formerly *Lactobacillus reuteri*^[32]^, which has immunomodulatory properties, including effects on tolerogenic immune responses by promoting the expansion of Tregs, intraepithelial lymphocytes, and ILCs^[33-38]^. *L. reuteri* is ideal for studying the effects of immunomodulatory bacteria in mice as lactobacilli are highly abundant in the mouse gut microbiome^[39]^, and *L. reuteri* shares stable evolutionary relationships with and are autochthonous to rodents^[40,41]^. However, only a few studies have examined the ability of this species to modulate the immune system in early life^[38,42-44]^, and the degree to which early life aberrations in immune development can be redressed by immunomodulatory bacteria is unclear.

The objectives of this study were to determine: (i) the relative importance of the prenatal and early postnatal period for the gut microbiome to influence the development of the immune system and (ii) whether *L. reuteri* can redress potential alterations in immune development resulting from delayed colonization of the gut microbiome. We achieved this through experiments in mice where the timing of microbial exposure was strictly controlled using gnotobiotic methodology. At the endpoint, the effects of microbial exposure at different time points on immune cell populations were determined using flow cytometry and complete blood count (CBC) with differential.

## METHODS

### Animals and animal husbandry

The mouse experiments were approved by the University of Alberta Research and Ethics Board under Animal Use Protocol 2410. GF Swiss Webster mice that were originally obtained from Taconic Biosciences were bred and housed in shoebox cages (3 to 5 mice per cage) in sterile flexible film isolators (Controlled Environment Products) at the Axenic Mice Research Unit at the University of Alberta. The GF status of the mice was assessed prior to starting the experiments and during the experiments by PCR using universal primers for the detection of the 16S rRNA gene (16S-8-F: 5’-AGAGTTTGATCCTGGCTCAG-3’; 16S-1391-R: 5’-GACGGGCGGTGWGTRCA-3’) using DNA extracted from fecal samples [Supplementary Figure 1A]. DNA extraction was performed using the QIAamp DNA Stool Mini kit (Qiagen) according to the manufacturer’s protocol, with the addition of a bead-beating step for homogenization. Results from the PCR were confirmed by culturing fecal samples in Wilkins-Chalgren (WC, Oxoid), yeasts and molds [YM, Beckton Dickinson (BD)], and brain heart infusion (BHI, BD) broth and on BHI 5% blood agar plates (BD) in both aerobic and anaerobic conditions at 37 °C for up to seven days [Supplementary Figure 1B and C]. An anaerobic chamber (Sheldon Manufacturing) with a gas mixture of 5% CO_2_, 5% H_2_, and 90% N_2_ or anaerobic gas packs (Mitsubishi Gas Chemical) and anaerobic jars (Mitsubishi Gas Chemical) were used for culturing in anaerobic conditions.

Experimental mice were kept in sterile isolators for the duration of the experiments. The mice had free access to sterile food and water. To establish the experimental groups, breeding pairs of GF mice were established to produce offspring. Female mice used for breeding were 10.4 (± 3.75) weeks old and male mice were 17.9 (± 8.15) weeks old at the time of breeding [mean (± standard deviation (SD)) weeks old]. Litters were weaned at three weeks of age (PND 21), and at this time, the mice were separated into shoebox cages based on their sex (3 to 5 mice per cage). At the time of weaning, the pups’ food was changed from an autoclavable breeder diet (LabDiet 5021, LabDiet) to an autoclavable maintenance diet (LabDiet 5010, LabDiet). Sterile cardboard huts (Ketchum) and cotton nestlets (Ancare) were provided for environmental enrichment. Animals were housed under 12:12 or 14:10 light:dark cycling conditions.

### Colonization of mice with a complex microbiome at different ages

To conventionalize the mice, diluted cecal contents [diluted 1:4 in sterile phosphate-buffered saline (PBS)] were dripped and rubbed onto the fur of the pups and dams or 4-week-old mice and dripped onto the surrounding bedding using a 1 mL syringe, as previously described^[45,46]^. This was done instead of an oral gavage to reduce the stress on the dam and because an oral gavage of newborn pups was not feasible. Since mice groom themselves regularly and are coprophagic, this will ensure highly efficient transmission of microbes among mice in the same cage. To ensure consistency in the treatment methods, the mice that were conventionalized within 48 h of birth (EC mice) or at PND 28 (DC mice) were treated with the cecal contents in the same manner. The cecal contents used as the inoculum in these experiments were aseptically harvested from healthy conventional adult C57BL/6 mice (from the University of Nebraska-Lincoln) and stored at -80 °C until use.

Successful colonization by the gut microbiome using this method was confirmed in EC mice one week after treatment by culturing fecal pellets from the parents of the pups on BHI blood agar plates and in WC, YM, and BHI broths [Supplementary Figure 1D]. Cultures were incubated at 37 °C in aerobic and anaerobic conditions for 48 h and monitored for growth.

### Colonization of mice with *L. reuteri*

*L. reuteri* R2lc was kindly provided by Jan-Peter van Pijkeren (University of Wisconsin-Madison)^[47]^. *L. reuteri* PB-W1 was isolated in our lab from the gut microbiome of an individual from Papua New Guinea^[48]^. Cultures for *L. reuteri* treatment of the mice were prepared by separately growing *L. reuteri* R2lc and *L. reuteri* PB-W1 on mMRS agar plates (55 g/L MRS broth powder (BD), 10 g/L D(+) maltose (Fisher Bioreagents), 5 g/L D(-) fructose (Fisher Chemical), 0.5 g/L L(-) cysteine (ACROS Organics), and 15 g/L agarose (Fisher Bioreagents)) for 48 h at 37 °C in anaerobic conditions. Subsequently, for each bacterium, a single colony was transferred into 10 mL of mMRS broth and incubated for 16 h at 37 °C in anaerobic conditions. These cultures were used to colonize the pups within 48 h of birth following the same procedure as for cecal contents [Supplementary Figure 1E and F]. The concentrations of the cultures used for treatment were 2.10 × 10^9^ (± 9.90 × 10^8^) CFU/mL (*n* = 2) and 1.60 × 10^9^ (± 4.36 × 10^8^) CFU/mL (*n* = 3) for *L. reuteri* PB-W1 and *L. reuteri* R2lc, respectively [mean (± SD) CFU/mL] [Supplementary Figure 1E and F]. Colonization by *L. reuteri* was confirmed one week after treatment by culturing fecal samples on mMRS agar plates and in mMRS broth [Supplementary Figure 1E and F]. Cultures were incubated at 37 °C for 48 h in anaerobic conditions and monitored for growth. The microbial density of these samples was 2.51 × 10^7^ (± 3.05 × 10^7^) CFU/mL (*n* = 2) and 1.17 × 10^8^ (± 3.27 × 10^7^) CFU/mL (*n* = 4) for *L. reuteri* PB-W1 and *L. reuteri* R2lc, respectively [mean (± SD) CFU/mL] [Supplementary Figure 1E and F].

### Mouse experiments

**Figure 1:**
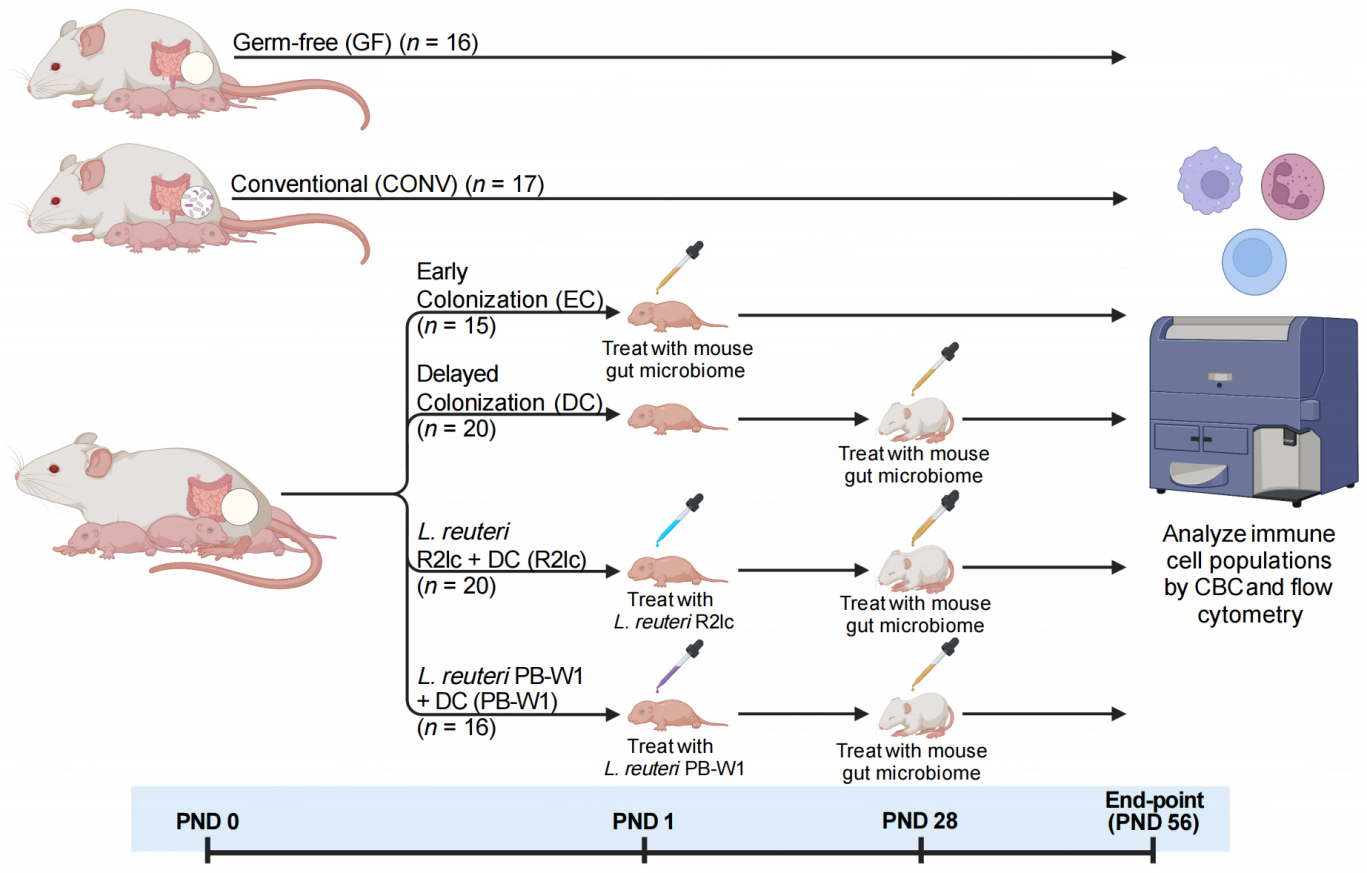
Experimental design of this study. Created with BioRender.com. PND: postnatal day.

The overall experimental design is shown in Figure 1. GF Swiss Webster mice (*n* = 16) were obtained from the breeding colony. Conventional Swiss Webster mice were pups born to dams colonized by a complex microbiota at the time of mating (CONV mice) (*n* = 17). To determine the importance of the timing of exposure to microbial signals for immune development, we compared CONV mice to mice born to GF dams that were conventionalized with a mouse gut microbiome either within 48 h of birth (early colonization; EC) (*n* = 15) or at four weeks of life (PND 28) (delayed colonization; DC) (*n* = 20).

To determine the ability of *L. reuteri* to redress the potential effects of delayed colonization of the gut microbiome on immune development, pups born to GF dams were mono-associated with either *L. reuteri* R2lc (the R2lc group) (*n* = 20) or *L. reuteri* PB-W1 (the PB-W1 group) (*n* = 16) within 48 h of birth, followed by conventionalization at four weeks of life (PND 28) [Figure 1].

Mice were euthanized at eight weeks of age for sample collection. Samples collected include spleens, mesenteric lymph node (MLN), and Peyer’s patch (PP) for the characterization of immune cell populations by flow cytometry and blood, where immune cells were analyzed by CBC. Two independent experiments, each with one litter per experimental group, were conducted. 

### Complete blood count with differential 

Whole blood was collected immediately after euthanasia and transferred to K_2_EDTA-coated microtainers (BD). Approximately 150 µL of whole blood was used for CBC, which was performed using the ADVIA 2120i Multi-Species Hematology System (Siemens Healthcare). It should be noted that some blood samples could not be analyzed due to a low volume of blood that was collected, which was not sufficient for analysis by CBC, or there was clotting, thus reducing the number of samples that could be included in the subsequent analysis.

### Flow cytometry

Harvested spleens, MLN, and PP were collected into 5 mL complete RPMI (RPMIc: RPMI 1640 media (Cytiva), 1% minimum essential medium (MEM) non-essential amino acids (Gibco), 1% sodium pyruvate (Gibco), 1% penicillin-streptomycin (Gibco), 1% L-glutamine (Gibco), 0.04% β-mercaptoethanol (Sigma-Aldrich), and 10% heat-inactivated bovine calf serum (BCS) (Sigma-Aldrich)) and kept on ice until processing. Cell suspensions from spleens were obtained by pushing the tissue through a 70 µm cell strainer. Red blood cells in the spleens were lysed with ACK lysis buffer (Gibco) for 3 min at room temperature. After three washes with 5 mL of cold RPMIc, isolated splenocytes were filtered through a 40 µm cell strainer and counted using the EVE automatic cell counter (NanoEnTek). Ninety-six well V-bottom plates (Greiner Bio-One) were subsequently seeded with 1.25 × 10^6^ cells per well. Cells were stained with a fixable viability dye [fixable viability stain 510 (BD) or fixable viability stain 620 (BD)] in PBS before staining for surface markers in staining buffer [1% BCS (Sigma-Aldrich) and 0.1% sodium azide (Fisher Chemical) in PBS, pH 7]. For intracellular markers, isolated splenocytes were stimulated with anti-CD3 (Invitrogen) and anti-CD28 (Invitrogen) overnight at 37 °C and 5% CO_2_. Brefeldin A (Invitrogen) was added for the last 4 h of incubation. Similarly, stimulated splenocytes were first stained with a fixable viability dye [fixable viability stain 620 (BD)] in PBS, followed by staining for surface markers in a staining buffer. Subsequently, the cells were fixed and permeabilized with the Foxp3/transcription factor staining buffer kit (Invitrogen) and stained for intracellular markers in permeabilization buffer (Invitrogen).

MLN and PP samples from every two mice were pooled prior to preparing cell suspensions to obtain sufficient cell numbers for analysis. MLN and PP were homogenized by grinding the tissue against a 70 µm cell strainer, followed by a single wash with 5 mL of cold RPMIc. Isolated immune cells from these tissues were only stained for surface markers, following the same protocol as the one used for the splenocytes.

The antibodies used in this experiment are listed in Table 1. The fixable viability dyes were diluted 1:1000 in PBS, and antibodies for surface markers were diluted 1:200 in staining buffer, while antibodies for intracellular markers were diluted 1:100 in permeabilization buffer.

**Table 1:** Antibodies and viability dyes that were used in this study

**Reagent**	**Source**	**Identifier**
Rat anti-mouse F4/80 (eFluor450, clone BM8)	Invitrogen	Cat. #: 48-4801-82; RRID: AB_1548747
Rat anti-mouse CD4 (Brilliant Violet 605, clone RM4-5)	BD Biosciences	Cat. #: 563151; RRID: AB_2687549
Armenian hamster anti-mouse CD11c (FITC, clone HL3)	BD Biosciences	Cat. #: 561045; RRID: AB_10562385
Rat anti-mouse NKp46 (CD335) (Alexa Fluor 647, clone 29A1.4)	BD Biosciences	Cat. #: 560755; RRID: AB_1727464
Rat anti-mouse CD45 (Alexa Fluor 700, clone 30-F11)	BD Biosciences	Cat. #: 560510; RRID: AB_1645208
Rat anti-mouse CD19 (APC-Cy7, clone 1D3)	BD Biosciences	Cat. #: 557655; RRID: AB_396770
Rat anti-mouse CD8a (Alexa Fluor 488, clone 53-6.7)	BD Biosciences	Cat. #: 557668; RRID: AB_396780
Mouse anti-mouse Foxp3 (Alexa Fluor 647, clone MF23)	BD Biosciences	Cat. #: 560402; RRID: AB_1645202
Rat anti-mouse CD25 (APC-Cy7, clone PC61)	BD Biosciences	Cat. #: 557658; RRID: AB_396773
Armenian hamster anti-mouse CD3 (clone 145-2C11)	Invitrogen	Cat. #: 16-0031-82; RRID: AB_468847
Syrian hamster anti-mouse CD28 (clone 37.51)	Invitrogen	Cat. #: 16-0281-82; RRID: AB_468921
Fixable viability stain 510	BD Biosciences	Cat. #: 564406; RRID: AB_2869572
Fixable viability stain 620	BD Biosciences	Cat. #: 564996; RRID: AB_2869636

APC: Allophycocyanin; BD: Becton Dickinson; FITC: fluorescein isothiocyanate.

Cells were acquired using the Attune NxT flow cytometer (Life Technologies Inc.). Flow cytometry data were analyzed using FlowJo V10 (BD). The total sample volume and thus all cells in the samples were collected and used for analysis. For spleen samples, the total number of cells acquired was 270,678 (± 71,973) cells for the EC group, 274,831 (± 123,169) for the DC group, 307,701 (± 84,488) for the R2lc group, 326,462 (± 97,939) for the PB-W1 group, 296,349 (± 84,258) for the CONV group, and 262,184 (± 70,018) for the GF group (mean ± SD). For MLN samples, the number of cells acquired was 256984 (± 178,480) for the EC group, 188,032 (± 135,703) for the DC group, 228,893 (± 102,984) for the R2lc group, 194,204 (± 164,940) for the PB-W1 group, 197,736 (± 64,694) for the CONV group, and 229,759 (± 143,648) for the GF group (mean ± SD). For PP samples, the number of cells acquired was 222,963 (± 120,598) for the EC group, 255,521 (± 185,485) for the DC group, 254,876 (± 89,067) for the R2lc group, 311,467 (± 128,167) for the PB-W1 group, and 280,866 (± 92,500) for the CONV group (mean ± SD). The overall gating strategy is shown in Supplementary Figure 2. Unstained samples, isotype controls, and Fluorescence Minus One (FMO) controls were used to set the gates.

### Principal components analysis

Principal components analysis (PCA) was performed using the “prcomp” function in R. PCA plots were generated using the fviz_pca_biplot function from the “factoextra” package^[49]^. To assess whether the distance between clusters was significantly different and to assess potential cage, experimental replicate, and sex effects, PERMANOVAs were performed using the adonis function from the vegan package in R^[50]^. The R code for these analyses is available from the DOI in the availability of data and materials section. Statistical significance was determined by a *P*-value of less than 0.05.

### Statistical analysis

To gain insight into the general role of the gut microbiota in shaping immune functions, GF and CONV mice were compared using an unpaired, two-tailed *t-*test. To determine the relative importance of the prenatal and early postnatal periods for microbial signals to modulate immune development, CONV, EC, and DC mice were compared using a one-way ANOVA. If the result of the one-way ANOVA was statistically significant, the Tukey-Kramer multiple comparisons test was performed. Comparing CONV and EC mice will indicate the effects of microbial signals in the prenatal period on immune development, and comparing EC and DC mice will indicate the influence of the gut microbiome in the early postnatal period on immune development. To determine the ability of the *L. reuteri* strains to redress alterations in immune development, the EC, DC, R2lc, and PB-W1 groups were compared using a one-way ANOVA. If the result of the one-way ANOVA was statistically significant, the Tukey-Kramer test was performed. Skewness and kurtosis values were assessed to determine the suitability of the data for these parametric statistical tests. No data transformations were performed prior to the statistical analyses. Reported *P*-values from multiple comparison tests are multiplicity-adjusted *P*-values. Statistical significance was determined by a *P*-value of less than 0.05. Statistical analyses were performed using GraphPad Prism V9 (GraphPad Software). Results from these statistical analyses are provided in Supplementary Tables 1, 2, and 3.

## RESULTS

### Experimental design

The overall study consisted of six experimental groups [Figure 1]. Conventional Swiss Webster mice (born to dams colonized by a complex mouse microbiota at the time of mating; CONV) (*n* = 17) and GF mice (*n* = 16) were included to establish how the immune system develops with and without a microbiota. To determine the importance of the timing of exposure to microbial signals for immune development, we compared immune phenotypes in CONV mice to mice born to GF dams that were conventionalized with a mouse gut microbiome either within 48 h of birth (early colonization; EC) (*n* = 15) or at four weeks of life (postnatal day (PND) 28) (delayed colonization; DC) (*n* = 20) [Figure 1]. To determine the ability of *L. reuteri* to redress the potential effects of delayed colonization of the gut microbiome on immune development, pups born to GF dams were mono-associated with either *L. reuteri* R2lc (the R2lc group) (*n* = 20) or *L. reuteri* PB-W1 (the PB-W1 group) (*n* = 16) within 48 h of birth, followed by conventionalization at four weeks of life (PND 28) [Figure 1], and immune phenotypes were compared to EC and DC mice. Mice were euthanized at eight weeks of age for sample collection. Samples collected include spleens, MLN, and PP for the characterization of immune cell populations by flow cytometry and blood, where immune cells were analyzed by CBC. Two independent experiments, each with one litter per experimental group, were conducted.

### Germ-free and conventional mice vastly differ in immune cell populations within the gut and systemically

**Figure 2:**
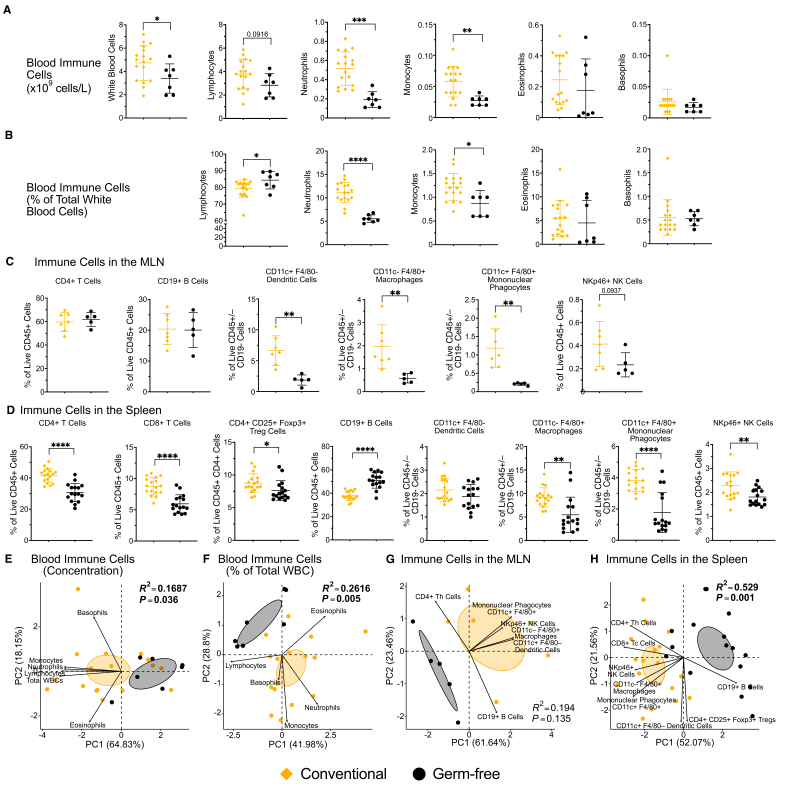
The gut microbiome affects immune cell populations in the blood, MLN, and spleen. Circulating immune cell populations in CONV and GF mice were measured by CBC. (A) The absolute concentration and (B) the proportion of total white blood cells of circulating immune cell populations in CONV and GF mice. Flow cytometry was used to characterize immune cell populations in the (C) MLN and (D) spleen of CONV and GF mice. Representative flow cytometry plots and the gating strategy can be found in Supplementary Figure 2. PCA of the (E) concentrations and (F) proportions of circulating immune cell populations and the proportions of immune cell populations in the (G) MLN and (H) spleen in GF and CONV mice. For (A), (B), (C), and (D), statistical significance was determined using unpaired, two-tailed t-tests. Symbols represent individual samples, and lines represent the mean ± standard deviation. For (E), (F), (G), and (H), ellipses represent the 95% confidence interval, each point represents one sample, and statistical significance was determined using PERMANOVA. Data are representative of two independent experiments. ^*^P < 0.05; ^**^P < 0.01; ^***^P < 0.001; ^****^P < 0.0001. See also Supplementary Tables 1 and 4.

To establish a baseline on how the gut microbiome affects the immune system and to determine our ability to confirm findings from research that spans several decades^[4,6-8,10]^, we first compared immune cell populations in the blood and lymphoid tissue in GF and CONV Swiss Webster mice. In the blood, we found that CONV mice had significantly greater concentrations of total white blood cells, neutrophils, and monocytes, with a trend for a greater concentration of lymphocytes compared to GF mice (*P* < 0.1) [Figure 2A]. In addition, there were significant differences in the proportion (percent of total white blood cells) of lymphocytes, neutrophils, and monocytes [Figure 2B].

To assess the influence of the gut microbiome on immune cell populations in lymphoid tissues, we assessed immune cell populations in the MLN and spleen of CONV and GF mice by flow cytometry. Due to a low number of events, it was not possible to assess immune cell populations in the PP of GF mice, which is in line with prior findings of impaired PP development in GF mice^[7]^. There were no significant differences in the proportions of CD4+ T cells and CD19+ B cells in the MLN of CONV and GF mice [Figure 2C]. However, the proportion of CD11c+ F4/80- dendritic cells and CD11c- F4/80+ macrophages, and CD11c+ F4/80+ mononuclear phagocytes were significantly greater in CONV mice compared to GF mice, and the proportion of CD4- NKp46+ natural killer (NK) cells tended to be greater in CONV mice (*P* < 0.1) [Figure 2C]. In the spleen, the proportions of nearly all immune cell populations identified significantly differed between GF and CONV mice. The proportion of CD4+ T cells, CD8+ T cells, CD4+ CD25+ Foxp3+ Tregs, CD11c- F4/80+ macrophages, CD11c+ F4/80+ mononuclear phagocytes, and CD4- NKp46+ NK cells were significantly greater in CONV mice, while the proportion of CD19+ B cells was lower in CONV mice compared to GF mice [Figure 2D]. Results from the statistical analyses for these comparisons are provided in Supplementary Table 1.

To quantify the effects of the gut microbiome on the immune system, we performed principal components analysis (PCA) and determined the effect sizes of the differences between the CONV and GF groups using PERMANOVA. This analysis showed significant differences between the CONV and GF groups for the concentrations (*R^2^* = 0.1687, *P* = 0.036) and proportions (*R^2^* = 0.2616, *P* = 0.005) of circulating immune cell populations, as well as for the proportions of immune cell populations in the spleen (*R^2^* = 0.529, *P* = 0.001) [Figure 2E, F and H]. There was no significant separation of these groups for immune cell populations in the MLN (*R^2^* = 0.194, *P* = 0.135) [Figure 2G]. PERMANOVA was also used to assess the importance of the treatment condition and confounders (experimental replicate, sex, and cage) on these findings. While there were statistically significant effects of experimental replicate and cage factors with respect to the proportion of circulating immune cells and immune cells in the spleen, the effect sizes are much smaller than the effect size of the treatment condition [Supplementary Table 4]. This indicates that these findings were primarily driven by the colonization status of the mice.

Overall, these findings show that 16.87% of the variation in the concentrations of circulating immune cells and 26.16% and 52.9% of the variation in the proportion of immune cell populations in the blood and spleen, respectively, can be explained by the colonization status of the mice, confirming the pronounced effect of the gut microbiome on the immune system.

### Microbial signals in the postnatal period are more important for immune system development than microbial cues in the prenatal period 

To assess the relative importance of the prenatal and early postnatal periods for the gut microbiome to influence immune development, we compared immune cell populations in the blood, spleen, MLN, and PP in mice born to conventional dams (CONV mice) and mice that were born to GF dams and conventionalized within 48 h of birth (early colonized; EC mice) or at four weeks of life (delayed colonized; DC mice) (for the experimental design see Figure 1). Comparisons between CONV and EC mice allowed for the determination of the effects of microbial cues in the prenatal period on immune development, while comparisons between EC and DC mice allowed for the determination of the effects of the gut microbiome in the early postnatal period on immune development.

**Figure 3:**
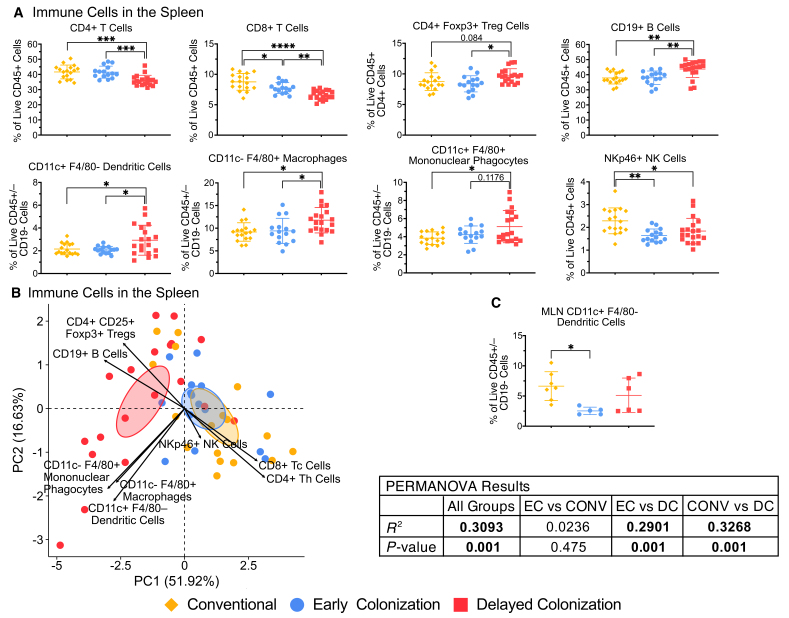
Comparison of immune cell populations in CONV, early colonized (EC), and delayed colonized (DC) mice. (A) Immune cell populations in the spleen of CONV, EC, and DC mice were characterized using flow cytometry; (B) PCA of the proportions of immune cell populations in the spleen; (C) CD11c+ F4/80- dendritic cells in the MLN of CONV, EC, and DC mice were assessed using flow cytometry. Representative flow cytometry plots and the gating strategy can be found in Supplementary Figure 2. For (A) and (C), statistical significance was determined using one-way ANOVA followed by the Tukey-Kramer multiple comparisons test. Symbols represent individual samples and lines represent the mean ± standard deviation. For (B), ellipses represent the 95% confidence interval, symbols represent individual samples, and PERMANOVAs were used to assess whether the clustering was significantly different. Data are representative of two independent experiments. *P < 0.05, **P < 0.01, ***P < 0.001. See also Supplementary Figures 3 and 4 and Supplementary Tables 2 and 5.

Most of the effects of these experimental conditions were detected in the spleen. Comparing CONV and EC mice revealed that only CD8+ T cells and NKp46+ NK cells in the spleen significantly differed [Figure 3A]. Other splenic immune cell populations did not significantly differ, and there was no significant separation of the CONV and EC groups by PCA (*R^2^* = 0.0236, *P* = 0.475) [Figure 3A and B]. In contrast, comparing EC and DC mice revealed altered proportions of CD4+ T cells, CD8+ T cells, CD4+ CD25+ Foxp3+ Tregs, CD19+ B cells, CD11c+ F4/80- dendritic cells, CD11c- F4/80+ macrophages, and a trend for CD11c+ F4/80+ mononuclear phagocytes (*P* = 0.1176) in the spleen [Figure 3A]. PCA showed a significant separation of these groups (*R^2^* = 0.2901, *P* = 0.001) [Figure 3B]. PERMANOVA was also used to assess the effects of confounders (experimental replicate, sex, and cage) on these findings. While there was a statistically significant effect of sex and the cage environment with respect to splenic immune cell populations, the effect sizes of the confounders (sex: *R^2^* = 0.0598; cage: *R^2^* = 0.0754) were substantially lower than the effect size of the treatment condition (treatment: *R^2^* = 0.3093) [Supplementary Table 5], indicating that these findings were mainly driven by the treatment condition. 

Except for a significantly greater proportion of CD11c+ F4/80- dendritic cells in the MLN of CONV mice compared to EC mice [Figure 3C], we found no statistically significant differences between CONV and EC mice or between EC and DC mice for any of the immune cell populations analyzed in the blood [Supplementary Figure 3], MLN or PP [Supplementary Figure 4], which was confirmed by PCA. The results from the statistical analyses for these comparisons are included in Supplementary Table 2.

This analysis showed that 29.01% of the variation in the proportions of splenic immune cell populations is explained by differences between the EC and DC groups, while only 2.36% of the variation is explained by differences between the EC and CONV groups. Overall, this indicates that exposure to microbial products in the early postnatal period is relatively more important than exposure in the prenatal period for immune development, but some immune cell populations, including CD8+ T cells and NKp46+ NK cells in the spleen and CD11c+ F4/80- dendritic cells in the MLN, are especially affected by microbial cues in the prenatal period.

### *Limosilactobacillus reuteri* treatment in early life partially restores alterations in immune development resulting from delayed colonization of the gut microbiome 

Our findings above revealed major differences in splenic immune cell populations between EC and DC mice [Figure 3A and B], indicating that delaying colonization of the gut microbiome impairs the development of immune cell populations in the spleen. We included two additional experimental groups to test whether a single treatment with *L. reuteri* immediately after birth is capable of rectifying the effects of delayed colonization of the gut microbiome on immune system development. One of the ways that *L. reuteri* can modulate immune system functions is through the activation of the aryl hydrocarbon receptor (AhR) by tryptophan metabolites^[36]^. In addition to this method of AhR activation, *L. reuteri* R2lc has been shown to strongly activate the AhR through products from a polyketide synthesis gene cluster^[47]^*.* Therefore, we utilized this strain to examine the effects of strong AhR induction in immune development. The incidence of asthma and allergies has increased greatly in the last hundred years in industrialized societies, and at the same time, certain bacteria from the gut microbiome have become rare or absent in industrialized people, including *L. reuteri*^[48]^. To assess the effects of bacteria that are more commonly found in non-industrialized people on immune development, we used *L. reuteri* PB-W1, which was isolated from a rural Papua New Guinean^[48]^. Therefore, we treated mice born to GF dams with either *L. reuteri* PB-W1 or *L. reuteri* R2lc within 48 h of birth. These mice remained mono-associated with their respective strain of *L. reuteri* throughout the preweaning period and subsequently were conventionalized with a complex mouse gut microbiome at four weeks of age (the experimental design is shown in Figure 1). Mono-association was confirmed by collecting feces from these mice one week after treatment and plating these samples on modified de Man, Rogosa, and Sharpe (mMRS) agar plates [Supplementary Figure 1E and F].

**Figure 4:**
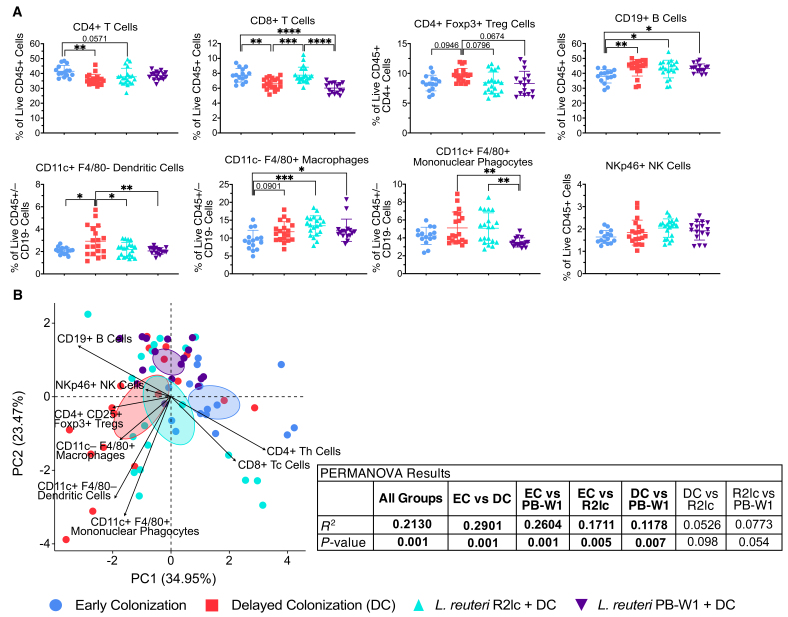
*Limosilactobacillus reuteri* restored splenic dendritic cell and regulatory T cell populations when colonization of the gut microbiome is delayed. (A) Flow cytometry was used to characterize immune cell populations in the spleen of EC, DC, R2lc, and PB-W1 mice. Representative flow cytometry plots and the gating strategy can be found in Supplementary Figure 2. Statistical significance was determined using a one-way ANOVA with the Tukey-Kramer multiple comparisons test. Symbols represent individual samples, and lines represent the mean ± standard deviation; (B) PCA of the proportions of immune cell populations in the spleen. Ellipses represent the 95% confidence interval, symbols represent individual samples, and PERMANOVA was used to assess whether the clustering was significantly different. Data are representative of two independent experiments. *P < 0.05, **P < 0.01, ***P < 0.001, ****P < 0.0001. See also Supplementary Tables 3 and 6.

We focussed this analysis on immune cell populations in the spleen as differences in immune cell populations between EC and DC mice were only detected in this tissue. Treatment with either the R2lc or PB-W1 strain of *L. reuteri* early in life restored the level of CD11c+ F4/80- dendritic cells in the spleen to a similar level as the EC group [Figure 4A]. The R2lc and PB-W1 groups also had a similar proportion of splenic CD4+ CD25+ Foxp3+ Tregs as the EC group, which was lower than the DC group (*F*_3,66_ = 3.070, *P* = 0.0338; DC *vs.* R2lc: *P* = 0.0796, DC *vs.* PB-W1: *P* = 0.0674) [Figure 4A]. As well, R2lc, but not PB-W1, restored the proportion of CD8+ T cells to the same level as the EC group [Figure 4A]. Conversely, the proportion of CD4+ T cells in the spleen was similar in the PB-W1 and EC groups but lower in the R2lc group compared to the EC group (*P* < 0.1) [Figure 4A]. There were also strain-specific effects with respect to the proportion of the CD11c+ F4/80+ mononuclear phagocytes population, which was lower in the PB-W1 group than in the R2lc and DC groups [Figure 4A]. However, treatment with either strain of *L. reuteri* did not restore the proportions of splenic CD19+ B cells and CD11c- F4/80+ macrophages to the level observed in the EC group, and they were at a similar level as the DC group [Figure 4A]. Results from the statistical analyses for these comparisons are provided in Supplementary Table 3.

The PCA confirms the partial restoration of splenic immune cell populations in the *L. reuteri*-treated groups as these groups while clustering closer to the DC group than the EC group, show some separation from the DC group (PB-W1 *vs.* EC: *R^2^* = 0.2604, *P* = 0.001; R2lc *vs.* EC: *R^2^* = 0.1711, *P* = 0.005; PB-W1 *vs.* DC: *R^2^* = 0.1178, *P* = 0.007; R2lc *vs.* DC: *R^2^* = 0.0526, *P* = 0.098) [Figure 4B]. An assessment of the effects of confounders (experimental replicate, sex, and cage) on these findings by PERMANOVA showed that while there was a statistically significant effect of sex and cage environment on these splenic immune cell populations, the effect sizes of these confounders were small (sex: *R^2^* = 0.0665; cage: *R^2^* = 0.0604) and less than that of the effect size of the treatment condition (*R^2^* = 0.2130) [Supplementary Table 6]. This indicates that the treatment condition was the primary factor in explaining the variation in the proportion of splenic immune cells.

Overall, these findings demonstrate that stable colonization from a single treatment with a single strain of *L. reuteri* soon after birth was able to partially restore splenic immune cell development when colonization of the gut microbiome was delayed, with some strain-specific effects with respect to CD4+ T cells, CD8+ T cells, and CD11c+ F4/80+ mononuclear phagocytes.

## DISCUSSION

**Figure 5:**
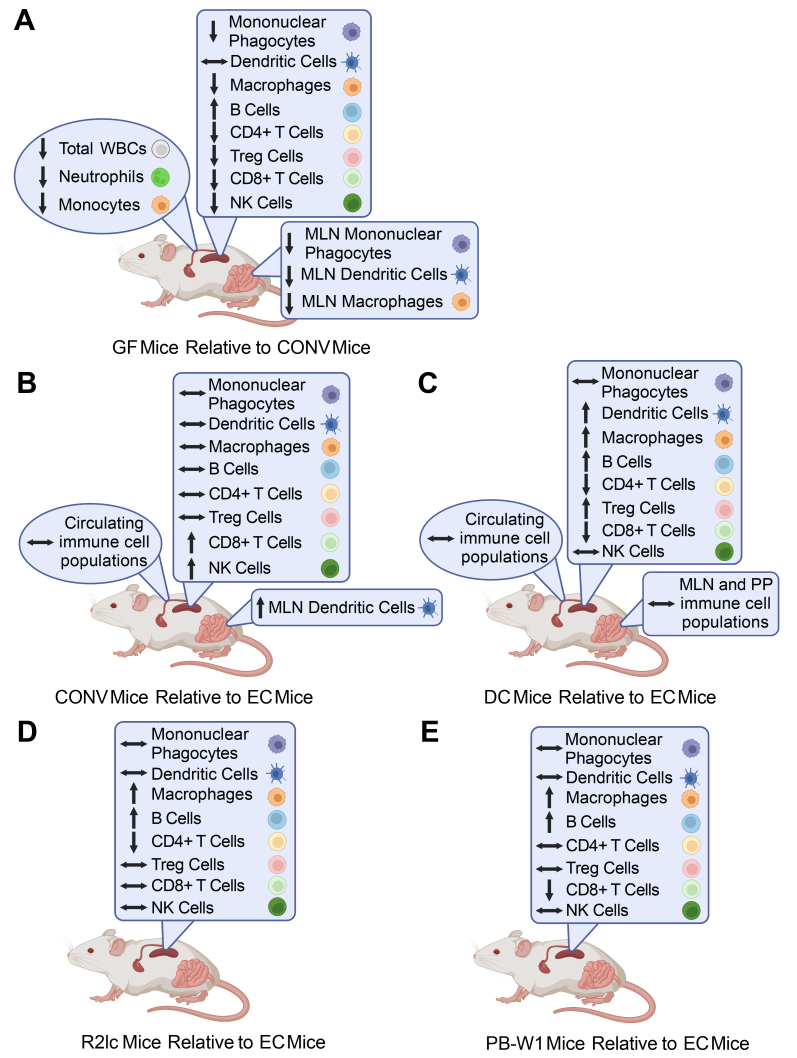
Summary of the effects of the gut microbiome and *L. reuteri* on immune cell populations in the blood, spleen, MLN, and PP. (A) Differences in immune cell populations in GF mice relative to CONV mice; (B-E) Differences in immune cell populations in (B) CONV mice; (C) mice conventionalized at four weeks of age (DC mice); (D) *L. reuteri* R2lc treated mice (R2lc mice); and (E) *L. reuteri* PB-W1 treated mice (PB-W1 mice) relative to mice conventionalized soon after birth (EC mice). Horizontal double-headed arrows indicate no difference between the groups, upward arrows represent a relatively greater amount, and downward arrows represent a relatively lesser amount. Created with BioRender.com.

In this study we demonstrate the importance of the gut microbiome for immune system development and confirm the immunological aberrations in germ-free mice [Figure 5A]. Our findings emphasize the importance of the early postnatal period, when compared to the prenatal period, for microbial signals to influence immune development [Figure 5B and C]. Moreover, we show that administration of *L. reuteri* early in life can restore the levels of splenic dendritic cells and Tregs to what was observed in EC mice [Figure 5D and E]. In addition, strain-specific effects of *L. reuteri* were observed with respect to the restoration of CD4+ T cells, CD8+ T cells, and CD11c+ F4/80+ mononuclear phagocytes in the spleen. However, not all immune cell populations were restored by *L. reuteri* treatment, which suggests that signals from multiple members of the neonatal gut microbiome are required for proper host development.

The only differences detected between EC and CONV mice were in the proportions of CD11c+ F4/80- dendritic cells in the MLN and CD8+ T cells, and NKp46+ NK cells in the spleen. A previous study found that compared to GF control mice, transient colonization of dams with *E. coli* HA107 during gestation resulted in an increased amount of certain innate immune cells in the intestinal lamina propria, including ILC3s and CD11c+ F4/80+ mononuclear cells, but had no effect on adaptive immune cell populations in the offspring^[26]^. Unlike this study, we did not observe a difference in CD11c+ F4/80+ mononuclear phagocytes in the MLN or PP between CONV and EC mice, which may be due to differences in the experimental approaches, including the sites in which immune cell populations were examined. However, overall, the findings from both studies demonstrate an effect of the maternal gut microbiota in the development of certain innate immune cell populations, especially in the intestines, and less of a role in the development of adaptive immune cell populations in the offspring.

While we found few differences between EC and CONV mice, there were profound differences in the immune cell populations examined in the spleen between EC and DC mice. Compared to EC mice, DC mice had a greater proportion of antigen-presenting cells (APCs) (CD19+ B cells, CD11c+ F4/80- dendritic cells, and CD11c- F4/80+ macrophages) and Tregs, as well as a reduced proportion of CD4+ and CD8+ T cell populations in the spleen. Our findings are relevant since microbial signals in the preweaning period promote proper immune system maturation and the development of tolerogenic responses against gut microbes, which ultimately results in an attenuation of inflammatory responses in various models of intestinal inflammation^[16,17]^. The greater proportion of splenic APCs in DC mice compared to EC mice may indicate a heightened sensitivity to foreign antigens since tolerance to foreign antigens, including the gut microbiome, was likely not generated early in life. The greater proportion of Tregs observed in DC mice compared to EC mice may be indicative of an altered immune environment in the spleen requiring greater control of immune responses and may partially explain the reduced proportions of other T cell populations compared to EC mice. However, since we did not measure absolute counts of these immune cell populations, the reduced proportion of T cell populations in DC mice may also simply be due to the expansion of APCs.

We also found that the concentrations and relative proportions of immune cell populations in the blood were not significantly different between EC and DC mice or EC and CONV mice. Given the aberrations in circulating immune cell populations observed in GF mice compared to CONV mice, this indicates that microbial signals from the gut can influence the levels of these immune cell populations regardless of the timing of microbial colonization.

While we observed greater proportions of CD11c+ F4/80- dendritic cells in the MLN in CONV mice compared to EC mice, there were no significant differences between EC and DC mice for any of the immune cell populations examined in the MLN or PP. This suggests that immune cell populations in these intestinal immune compartments do not require microbial signals specifically in the preweaning period for their development. Others have found that conventionalizing GF mice at five weeks of life can restore intestinal macrophage populations to levels observed in specific pathogen-free (SPF) mice^[51]^. However, Olszak *et al.* found that colonization of the gut microbiome after weaning (at five weeks of age) was not sufficient to restore the proportion of invariant natural killer T cells in the colonic lamina propria to levels observed in SPF mice^[12]^. Taken together, this suggests that while the gut microbiome influences the development of immune cell populations in the gut (as we observed by comparing GF and CONV mice), only certain immune cell populations in the gut require microbial signals in the early postnatal period for their proper development. 

Given the consequences of delaying colonization of the gut microbiome on immune development, we explored strategies to redress alterations in immune system development associated with delayed colonization of the gut microbiome using strains of a bacterial species (*L. reuteri*) with well-known immunomodulatory properties^[33-36,38,52]^ and evolutionary history with rodents^[40,41]^. Treatment with *L. reuteri* R2lc or *L. reuteri* PB-W1 soon after birth normalized the proportion of splenic CD11c+ F4/80- dendritic cells and CD4+ CD25+ Foxp3+ Tregs to a similar level as observed in EC mice. In addition, strain-dependent effects of *L. reuteri* on splenic T cell subsets were also observed, including the normalization of CD8+ T cells to what was observed in EC mice by *L. reuteri* R2lc but not by *L. reuteri* PB-W1. These strain-specific effects may be due to the potent activation of the AhR by *L. reuteri* R2lc^[47]^. *L. reuteri* can produce AhR ligands derived from tryptophan metabolism, such as indole and indole-derivative compounds, which promote intraepithelial lymphocyte differentiation^[33]^, stimulate IL-22 production^[36,52]^, and the expansion of ILC3s in the small intestine^[36]^. However, the R2lc strain of *L. reuteri* also produces an AhR ligand through a set of genes involved in polyketide synthesis, which more strongly activates the AhR than other strains of *L. reuteri*^[47]^. Thus, the strong activation of the AhR by *L. reuteri* R2lc may be one reason for the differences observed between the *L. reuteri* R2lc- and *L. reuteri* PB-W1-treated groups. Further investigations are warranted to determine the mechanisms underlying the effects of *L. reuteri* treatment in early life on immune development. Not all immune cell populations examined were normalized by *L. reuteri* to levels observed in EC mice, including CD19+ B cells and CD11c- F4/80+ macrophages, which suggests that colonization by a single immunomodulatory bacterium in early life is not sufficient to completely restore immune system development. Thus, synergistic signals from multiple members of the gut microbiome in the neonatal period are likely needed to facilitate proper immune system development.

We acknowledge the limitations of the models that we used in our experiments, which constitute extreme situations, with the rationale to establish proof of principles. Delayed microbiome colonization was used to examine the potential effects of perturbations to the neonatal gut microbiome in a critical period of life for immune development. We acknowledge that this does not recapitulate disruptions to the neonatal gut microbiome that would occur in humans, for example, due to Caesarean section (C-section) delivery or antibiotic exposure. Future research should assess host development in conventional mice exposed to antibiotics early in life or born by C-section. Since C-section delivery and antibiotic exposure in infancy have been associated with an increased risk of developing certain immune-mediated diseases^[53,54]^, future research should capitalize on murine disease models to directly examine the effects of disruptions to the neonatal gut microbiome on the development of disease pathologies. Clearly, the mono-association of *L. reuteri* for the first four weeks of life also constitutes an extreme situation. However, it is important to consider that when highly adapted gut microbes are administered to human infants, specific strains, such as *Bifidobacterium longum* subsp. *infantis*, can constitute around 80% of the gut microbiome, resembling mono-associated status^[55]^. Given that *B. longum* subsp. *infantis* has been shown to modulate immune development in human infants^[56]^; the mouse models established in our research are valuable to establish the mechanisms by which microbial cues shape early life immune development.

Both pregnancy and infancy are important periods for host development and for the assembly of the gut microbiome, and perturbations to both the maternal and infant gut microbiome in these periods may have lasting effects on the physiological development and health of the host. The results from this study indicate that the early postnatal period is relatively more important than the prenatal period for microbial signals to modulate immune development. In light of evidence indicating that the healthy mammalian fetus is protected from direct microbial stimuli^[57]^, our findings demonstrate that the importance of indirect microbial exposure (through metabolites or microbial products) to the fetus on later-life immune status is relatively much smaller than that of direct microbial stimuli after birth. This work, therefore, emphasizes the focus on the early postnatal period for microbiome modulation to aid immune system development. Specifically, our findings on the effects of *L. reuteri* demonstrate the value of using co-evolved gut symbionts as microbial-based therapeutics early in life to redress the effects of perturbations on the neonatal gut microbiome.

## DECLARATIONS

### Availability of data and materials

Resource availability: requests for further information and resources should be directed to the lead contact, Jens Walter (jenswalter@ucc.ie); Materials availability: this study did not generate any new unique reagents; Data and code availability: Code for the PCA and PERMANOVA analyses are publicly available at the time of publication (https://doi.org/10.5281/zenodo.6970229); All data will be made available by the lead contact upon request; Any additional information required to reanalyze the data presented in this paper is available from the lead contact upon request.

### Copyright

© The Author(s) 2023.
