# Supplementary material for: The importance of the timing of microbial signals for perinatal immune system development
Source: Microbiome Res Rep. 2023 Apr 15;2(2):11. doi: 10.20517/mrr.2023.03 (PMC10688825; doi:10.20517/mrr.2023.03)
Supplement: Supplementary file 1 [file mrr-2-2-11-SupplementaryMaterials.zip › SupplementaryMaterials.pdf]

Supplementary Figures

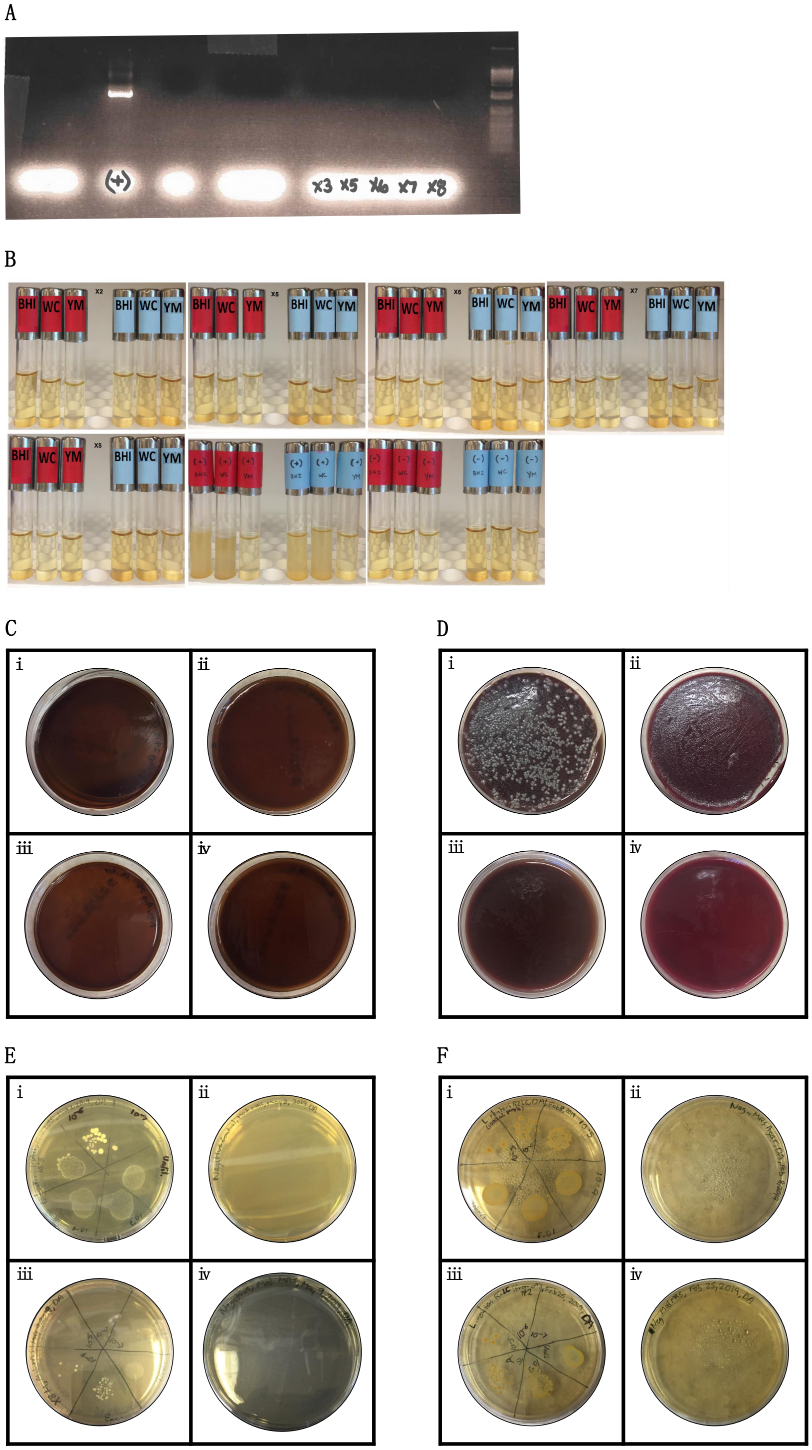

Supplementary Figure 1. Microbiological analysis to confirm germ-free status, colonization with a complex mouse gut microbiome, and colonization with *L. reuteri* PB-W1 or *L. reuteri* R2lc.

To confirm the GF status of the mice prior to starting the experiments, feces were collected from mice from each group (housed in isolators labelled X2, X3, X5, X6, X7, and X8) and suspended in PBS. (A) DNA was extracted from these samples and PCR using universal 16S rRNA primers was used to confirm the GF status of the mice ("(+)" refers to the positive control which in this case was feces from a conventional mouse). As well, feces were cultured in (B) BHI, WC, and YM broth and (C) streaked onto blood agar plates to confirm the GF status of the mice. Feces were collected from mice from the GF group prior to the start of the experiment were diluted in PBS and then streaked onto blood agar plates and incubated in (i) aerobic (red labelled tubes) or (ii) anaerobic conditions (blue labelled tubes). Negative control samples were included where sterile PBS was streaked on blood agar plates and incubated in (iii) aerobic or (iv) anaerobic conditions. (D) To confirm colonization of the gut microbiome after dripping cecal contents onto the fur of the mice, feces collected from mice from the EC group one week after treatment were diluted in PBS and then streaked onto a blood agar plate and incubated in (i) aerobic and (ii) anaerobic conditions. Negative control samples were included where sterile PBS was streaked on blood agar plates and incubated in (iii) aerobic and (iv) anaerobic conditions. (E) To determine microbial density, samples from the (i) *L. reuteri* PB-W1 culture used for treatment and (iii) feces from PB-W1 mice collected one week after treatment were plated on modified deMan, Rogosa, Sharp (mMRS) agar plates and incubated for 48 hours in anaerobic conditions. (ii, iv) Negative controls were included along with each sample. (F) Similarly, for *L. reuteri* R2lc, microbial density was determined by plating samples from the (i) *L. reuteri* R2lc culture (with its characteristic orange coloured colonies) used for treatment and (iii) feces from R2lc mice collected one week after treatment on mMRS agar plates and incubated for 48 hours in anaerobic conditions. (ii, iv) Negative controls were included along with each sample. Related to STAR Methods.

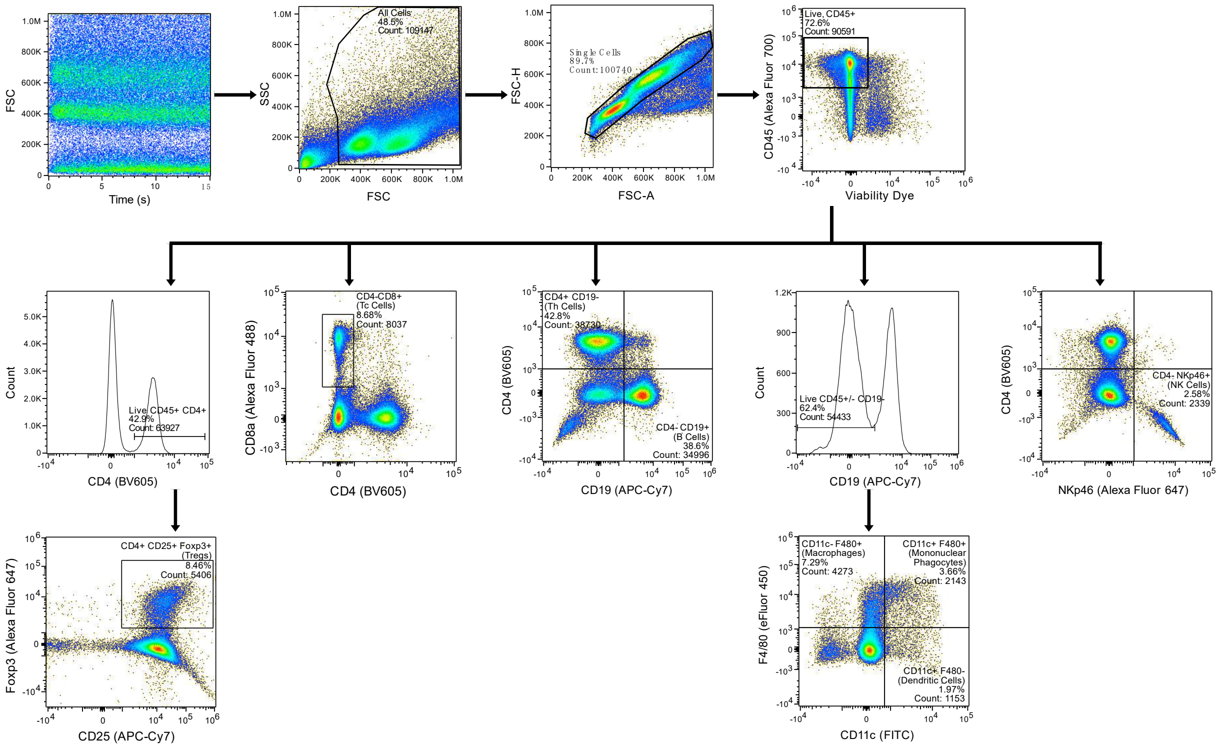

Supplementary Figure 2. Gating strategy for characterizing immune cell populations by flow cytometry.

Representative flow cytometry plots of splenic immune cell populations from a conventional mouse to illustrate the gating strategy used to characterize immune cell populations in the MLN, PP, and spleens. Unstained samples, isotype controls, and FMO controls were used to set the gates. The fixable viability stain used was either fixable viability stain 510 or fixable viability stain 620. Percentages shown are the percent of the parent population. Related to STAR Methods and Figures 2, 3, and 4.

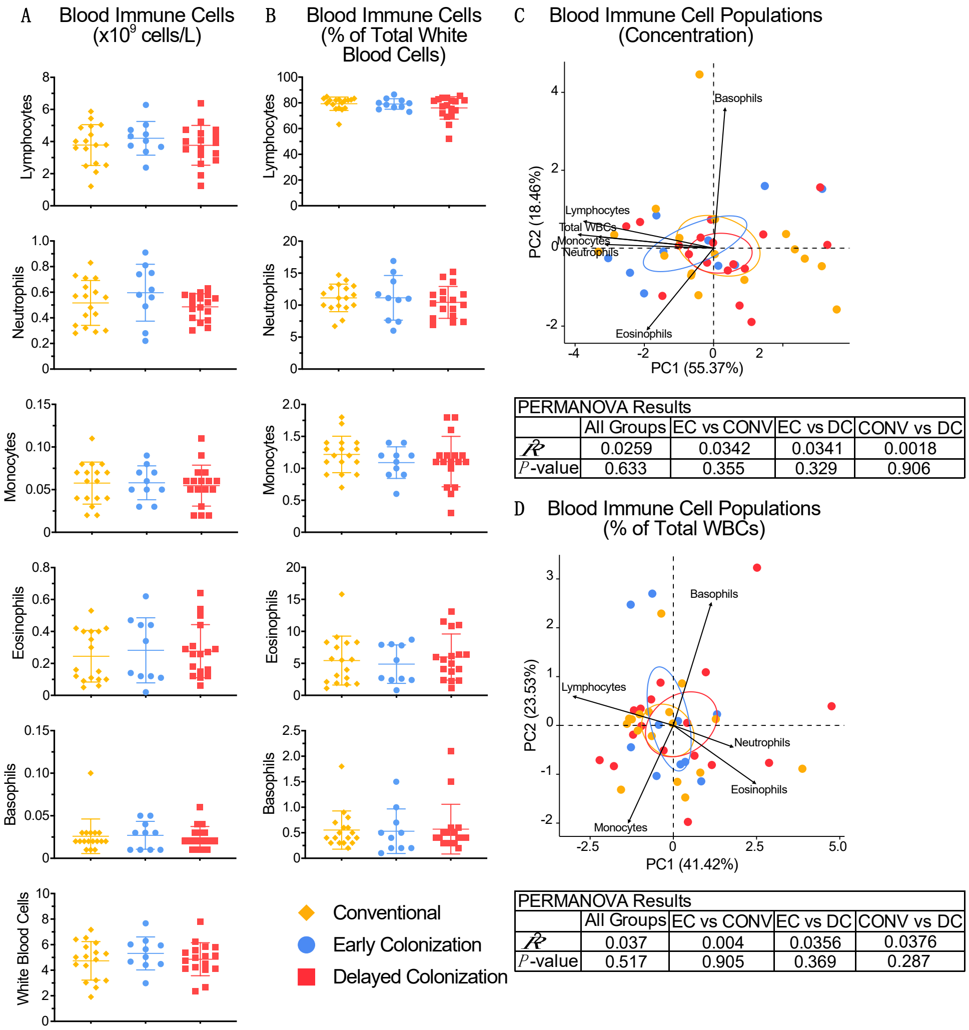

Supplementary Figure 3. Circulating immune cell populations in conventional (CONV), early colonization (EC), and delayed colonization (DC) mice.

Circulating immune cell populations in CONV, EC, and DC mice were measured by complete blood count and the (A) concentrations and (B) proportion of total white blood cells are shown. PCA for the (C) concentration and (D) proportion of circulating immune cells. For A, significance was determined using a one-way ANOVA followed by the Tukey-Kramer test for multiple comparisons where appropriate. Symbols represent individual samples and lines represent the mean ± standard deviation. For B, ellipses represent the 95% confidence interval, symbols represent individual samples, and PERMANOVA was used to assess whether the clustering was significantly different. Data are representative of two independent experiments. Related to Figure 3.

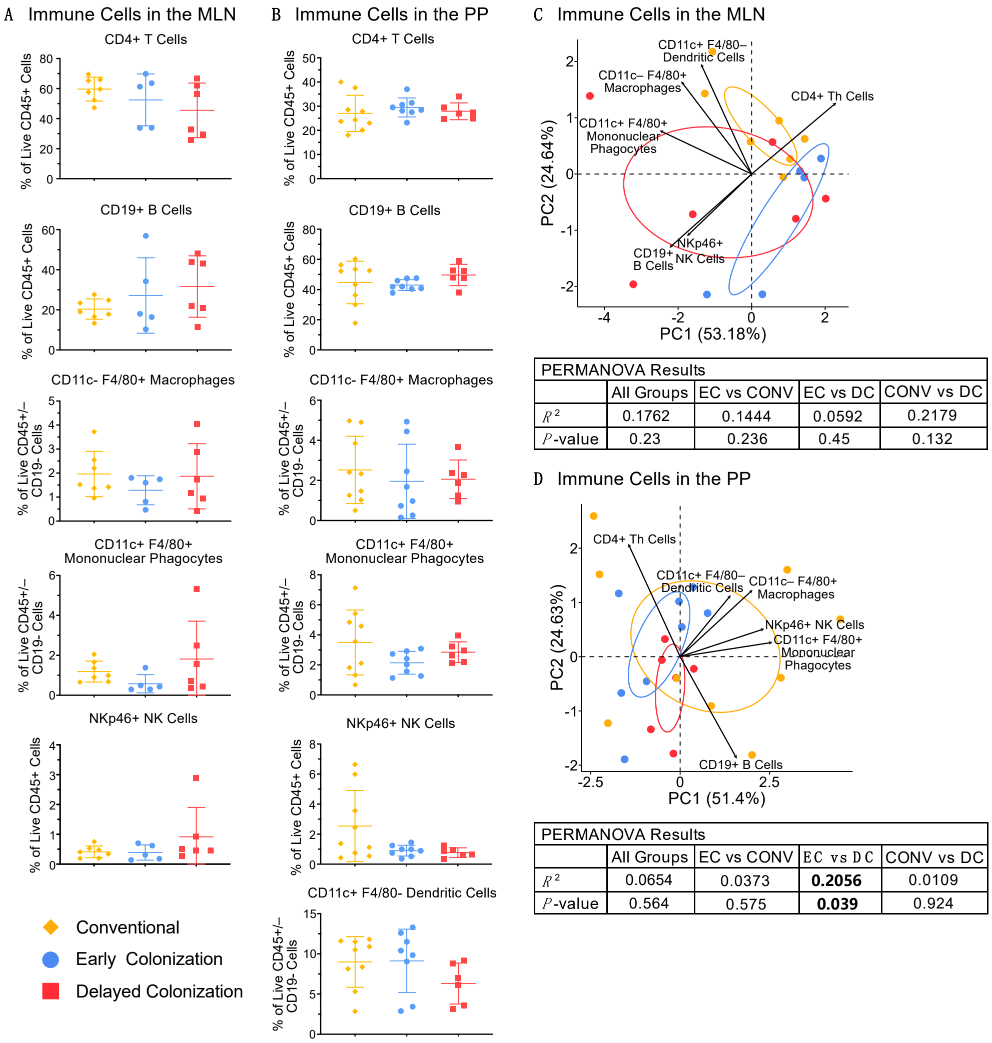

PERMANOVA Results	All Groups	EC vs CONV	EC vs DC	CONV vs DC
R^2	0.1762	0.1444	0.0592	0.2179
P-value	0.23	0.236	0.45	0.132

PERMANOVA Results	All Groups	EC vs CONV	EC vs DC	CONV vs DC
R^2	0.0654	0.0373	**0.2056**	0.0109
P-value	0.564	0.575	**0.039**	0.924

Supplementary Figure 4. Immune cell populations in the MLN and PP of conventional (CONV), early colonization (EC), and delayed colonization (DC) mice.

Immune cell populations in the (A) MLN and (B) PP of CONV, EC, and DC mice were characterized using flow cytometry. Representative flow plots and the gating strategy for this analysis can be found in Supplementary Figure S2. Statistical significance was determined using one-way ANOVA followed by the Tukey-Kramer test for multiple comparisons where appropriate. Symbols represent individual samples and lines represent the mean ± standard deviation. PCA of the proportions of immune cell populations in the (C) MLN or (D) PP. Ellipses represent the 95% confidence interval, symbols represent individual samples, and PERMANOVAs were used to assess whether the clustering was significantly different. Data are representative of two independent experiments. Related to Figure 3.

Supplementary Tables

Supplementary Table 1. Summary of the statistical analyses for the comparisons of GF and CONV mice. Related to Figure 2.

Supplementary Table 2. Summary of the statistical analyses for the comparisons of CONV, EC, and DC mice. Related to Figure 3.

Supplementary Table 3. Summary of the statistical analyses for the comparisons of EC, DC, R2lc, and PB-W1 mice. Related to Figure 4.

Supplementary Table 4. Analysis of the effects of potential confounders and their interactions with the treatment condition by PERMANOVA for circulating and splenic immune cell populations in the GF and CONV groups. Bolded values are statistically significant. Related to Figure 2.

	Circulating Immune Cells (Concentration)		Circulating Immune Cells (Percent of Total White Blood Cells)		Splenic Immune Cells	
Factor	R^2	*P*-value	R^2	*P*-value	R^2	*P*-value
Treatment	**0.1687**	**0.0210**	**0.2616**	**0.0010**	**0.529**	**0.001**
Cage	0.0066	0.6450	**0.1346**	**0.0230**	**0.1074**	**0.003**
Treatment:Cage	**0.3134**	**0.0040**	0.0107	0.6650	**0.0443**	**0.041**
Experiment	0.0540	0.1550	**0.1467**	**0.0250**	**0.1098**	**0.001**
Treatment:Experiment	**0.2379**	**0.0090**	-0.0076	0.9950	**0.0522**	**0.018**
Sex	0.0437	0.2760	0.0512	0.2370	0.0380	0.104
Treatment:Sex	0.0812	0.1340	0.0063	0.8050	0.0085	0.507

Supplementary Table 5. Analysis of the effects of potential confounders and their interactions with the treatment condition by PERMANOVA for immune cell populations in the CONV, EC, and DC groups. Statistically significant values are bolded. Related to Figure 3.

	Circulating Immune Cells (Concentration)		Circulating Immune Cells (Percent of Total White Blood Cells)		Splenic Immune Cells	
Factor	R^2	*P*-value	R^2	*P*-value	R^2	*P*-value
Treatment	0.0259	0.5520	0.0370	0.5200	**0.3093**	**0.001**
Cage	0.0348	0.1880	0.0641	0.0840	**0.0754**	**0.003**
Treatment:Cage	**0.1571**	**0.0280**	0.0386	0.4740	**0.1311**	**0.002**
Experiment	0.0625	0.0770	0.0702	0.0510	0.0367	0.057
Treatment:Experiment	0.1123	0.0670	0.0289	0.6030	**0.1681**	**0.001**
Sex	0.0134	0.4740	0.0063	0.7700	**0.0598**	**0.026**
Treatment:Sex	0.0959	0.1250	0.0351	0.5280	0.0198	0.509

Supplementary Table 6. Analysis of the effects of potential confounders and their interactions with the treatment condition by PERMANOVA for immune cell populations in the EC, DC, R2lc, and PB-W1 groups. Statistically significant values are bolded. Related to Figure 4.

	Circulating Immune Cells (Concentration)		Circulating Immune Cells (Percent of Total White Blood Cells)		Splenic Immune Cells	
Factor	R^2	*P*-value	R^2	*P*-value	R^2	*P*-value
Treatment	**0.1462**	**0.0200**	**0.1708**	**0.0050**	**0.2130**	**0.001**
Cage	0.0232	0.1960	0.0268	0.1750	**0.0604**	**0.001**
Treatment:Cage	**0.1299**	**0.0390**	0.0431	0.4450	**0.1956**	**0.001**
Experiment	0.0162	0.2790	0.0289	0.1510	**0.0576**	**0.003**
Treatment:Experiment	**0.1385**	**0.0290**	0.0445	0.4550	**0.1930**	**0.001**
Sex	0.0079	0.5150	0.0064	0.6690	**0.0665**	**0.001**
Treatment:Sex	0.0530	0.3630	0.0263	0.7320	0.0165	0.852
